# An improved tortuosity measurement method combining curvature-based, breadth-first search and Euclidean distance for retinal image analysis

**DOI:** 10.7717/peerj.20561

**Published:** 2026-01-06

**Authors:** Nur Asyiqin Amir Hamzah, Wan Mimi Diyana Wan Zaki, Aziah Ali

**Affiliations:** 1 Centre of Advanced Analytics, Faculty of Engineering and Technology, Multimedia University, Jalan Ayer Keroh Lama, Melaka, Malaysia; 2 Faculty of Engineering and Built Environment, Universiti Kebangsaan Malaysia, Bangi, Selangor, Malaysia; 3 Center for Image and Vision Computing, Faculty of Computing and Informatics, Multimedia University, Cyberjaya, Selangor, Malaysia

**Keywords:** Retinal vascular tortuosity, Artery-vein separation, Fundus image analysis, Curvature-based metrics, Euclidean distance, Field of view (FOV), Glaucoma, Diabetic retinopathy

## Abstract

Retinal vascular tortuosity is a clinically relevant biomarker linked to systemic and ocular diseases; however, its quantitative assessment particularly the distinction between arteries and veins remains underexplored in both healthy and pathological conditions. This study investigates tortuosity behavior using three publicly available retinal fundus image datasets: Digital Retinal Images for Vessel Extraction (DRIVE), High-Resolution Fundus (HRF), and Labelled Eye fundus Segmentation-Artery Vein (LES-AV). A standardized analytical pipeline combining curvature-based metrics, breadth-first search (BFS) and Euclidean distance was applied following vessel segmentation, artery-vein separation, skeletonization, and optic disc-based tracing. BFS algorithm was utilized for vessel path tracing, chosen for its robustness and suitability in navigating complex vascular structures with high reproducibility. Five comparative analyses were performed: artery *vs*. vein tortuosity; healthy *vs*. diseased eyes (glaucoma and diabetic retinopathy); and cross-dataset comparisons under both healthy and pathological conditions. Across all datasets, veins consistently exhibited higher tortuosity than arteries in healthy eyes, confirmed by large effect sizes (Cohen’s d), despite weak correlation between the two vessel types. Diabetic retinopathy cases showed an amplified artery-vein separation, suggesting disease-induced vascular changes. Glaucomatous eyes exhibited mixed patterns, with HRF artery-vein differences maintained and LES-AV showing diminished separation, partly due to non-normal data distribution. A key finding highlights the significant influence of imaging characteristics especially field of view (FOV) on tortuosity measurement. Datasets with a wider field of view, such as DRIVE and HRF (45° FOV, optical disc (OD) positioned nasally), captured more peripheral, tortuous vessels and reported higher tortuosity values. In contrast, the LES-AV dataset with a narrower 30° FOV and centered optic disc, resulted in lower tortuosity measurements and weaker artery-vein differentiation. These anatomical and technical differences emphasize the need to handle FOV and orientation settings when designing or comparing tortuosity-based diagnostic studies. In conclusion, retinal vascular tortuosity appears to be a vessel-specific feature which is robust and capable of capturing both pathological and variations in dataset. The findings support its integration into automated diagnostic frameworks and highlight the importance of standardizing imaging parameters particularly FOV and anatomical orientation in future retinal biomarker research.

## Introduction

Abnormalities in the morphology of retinal blood vessels have been widely associated with various cardiovascular, cerebrovascular, and systemic health conditions. Recent studies suggest that changes in retinal vasculature can serve as early indicators of disease onset, positioning retinal imaging particularly fundus imaging as a valuable tool for non-invasive, early diagnosis (Baumann, Burkhardt & Heemann, 2014; Grunwald et al., 2014; Amir Hamzah et al., 2024; Yau et al., 2011; Yip et al., 2017; Zhang & Deng, 2024). The retinal vasculature provides a unique window into microvascular health, as it reflects systemic circulatory changes observable through routine ophthalmic screening (Farrah et al., 2020). Its accessibility *via* fundus imaging allows for the early detection of vascular alterations that may signal underlying or progressing disease processes.

Several retinal vascular parameters have been explored as potential biomarkers, including central retinal artery equivalent (CRAE) and central retinal vein equivalent (CRVE), fractal dimensions, vessel tortuosity, and branching angles. These parameters have demonstrated associations with systemic disease progression, particularly in relation to cardiovascular and kidney dysfunction (Bao et al., 2015; Grunwald et al., 2012).

Among these, retinal vessel tortuosity which is a measure of the curvature or “twistiness” of blood vessels, has emerged as a clinically meaningful but underutilized feature (Mustafar et al., 2023). Retinal vascular tortuosity refers to the excessive bending and twisting of blood vessels in the retina, which can be an indicator of various systemic and ocular diseases, such as diabetic retinopathy, hypertensive retinopathy, and retinopathy of prematurity (Chuang & Handayani, 2023; Hervella et al., 2024; Wang et al., 2021).

Increased tortuosity may reflect vessel wall remodeling, hemodynamic stress, or early vascular damage due to chronic conditions such as diabetic retinopathy, glaucoma, and hypertension. Despite its clinical relevance, current tortuosity measurement methods vary widely in technique, often lack reproducibility, and frequently do not differentiate between arteries and veins. Furthermore, few studies have examined tortuosity across diverse datasets or compared its variation between healthy and diseased retinal images (O’Neill et al., 2020; Sasongko et al., 2012).

Recent literature reflects an evolving landscape in tortuosity measurement techniques. Wang et al. (2021) introduced a subdivision-based algorithm demonstrating robust prediction of diabetic outcomes, yet its application outside diabetic cohorts has not been fully validated. Hervella et al. (2024) introduced an explainable artificial intelligence (AI) framework integrating deep learning with clinical validation, achieving human-comparable performance. However, this approach depends heavily on the annotated ground truth and may not generalize well across datasets with different imaging conditions. Chuang & Handayani (2023) proposed a combined parameter using arc length, chord length, and angular values, yielding strong correlation with expert assessments. Nevertheless, their method may be limited in its ability to handle highly tortuous vessels or segmentation noise.

Curvature-based methods, eccentricity metrics, and U-COSFIRE filters have also been widely applied to differentiate vessel types and diagnose conditions like retinopathy of prematurity (Chen et al., 2020; Ramachandran et al., 2020). These methods often rely on precise segmentation and may struggle with low-quality images or overlapping vessels. Integration of anatomical features, such as distance to the optic disc and artery-vein classification, has further refined tortuosity (Khanal & Estrada, 2023; Ramos et al., 2020) though such approaches may suffer from computational complexity and dependency on accurate anatomical annotations.

Fully automated methods using vessel topology (Khanal & Estrada, 2023) offer comprehensive vascular feature analysis, but they often lack interpretability and clinical transparency. Differential geometry approaches (Manjunatha, Koti & Sheshadri, 2019) and image processing-based classification models (Chetia & Nirmala, 2019) demonstrate promising performance, yet these are limited by fixed rule sets and may not adapt well to diverse vessel patterns. Eccentricity-based quantification (De Zoysa et al., 2018) showed significant differences among disease stages, but its sensitivity to morphological artifacts warrants further validation.

Many studies do not explicitly report the field of view (FOV) used during image acquisition, yet this parameter can significantly influence tortuosity measurements due to the inclusion or exclusion of peripheral vessels. For example, studies utilizing clinical datasets such as the Retinal Vessel Tortuosity Dataset (RET-TORT) often operate on a 50° FOV, which captures wider retinal areas. In contrast, standard datasets like Digital Retinal Images for Vessel Extraction (DRIVE) and High-Resolution Fundus (HRF) typically provide images at a 45° FOV, whereas Labelled Eye fundus Segmentation-Artery Vein (LES-AV) offers a narrower 30° centered view. These variations affect the visibility of tortuous peripheral vessels and should be carefully considered when interpreting cross-study comparisons.

This study is based on the hypothesis that retinal vascular tortuosity demonstrates vessel-specific and disease-dependent variation that can be effectively quantified using a reproducible pixel-level path tracing approach. Additionally, imaging characteristics such as FOV and anatomical orientation significantly affect the tortuosity measurement and must be considered when developing robust, comparative, and diagnostic vascular analysis frameworks. The contributions of this work include:

- Proposing a reproducible, length-based tortuosity measurement method grounded in pixel-level vessel path tracing
- Performing artery-vein-specific tortuosity analysis using three publicly available datasets (DRIVE, HRF, and LES-AV)
- Comparing tortuosity characteristics across healthy and non-healthy (diabetic retinopathy and glaucoma) images
- Evaluating the effect of image acquisition differences, such as FOV, on tortuosity across datasets

The abbreviations used throughout this manuscript are defined in Table 1 for clarity.

**Table 1:** List of abbreviations.

AV	Artery and vein	HRF	High-Resolution Fundus
BFS	Breadth-First Search	ISODATA	Iterative Self-Organizing Data Analysis
CRAE	Central Retinal Arteriolar Equivalent	LAB	Lightness and Color Channels
CRVE	Central Retinal Venular Equivalent	LES-AV	Labelled Eye fundus Segmentation - Artery Vein
DR	Diabetic Retinopathy	OD	Optic Disc
DRIVE	Digital Retinal Images for Vessel Extraction	RBV	Retinal Blood Vessel
FOV	Field of View	RGB	Red, Green, Blue (color space)
HSV	Hue, Saturation, Value	ROI	Region of Interest

## Related works

Retinal vascular tortuosity has been studied through a variety of computational techniques over the past decade, reflecting its potential as a biomarker for systemic and ocular diseases. Nonetheless, the number of studies dedicated specifically to vessel tortuosity remains relatively limited compared to broader retinal image analysis research. This restricts comprehensive benchmarking and highlights the need for further exploration, particularly across diverse disease conditions and imaging modalities. However, there remains considerable variability in the methods, datasets, and validation approaches used. This section provides an overview of key studies and summarizes their methodologies, findings, and limitations.

Several studies have applied curvature-based and geometric analysis methods for tortuosity quantification. For instance, Wang et al. (2021) proposed a multiple subdivision-based algorithm that incorporates local and global curvature features to predict diabetic outcomes. The method achieved a strong correlation with expert grading (Spearman’s $\rho > 0.93$) and demonstrated high diagnostic performance ( $AUC = 0.96$), though it was limited to diabetic cohorts. Chuang & Handayani (2023) introduced a combined parameter method that calculates tortuosity based on arc length/chord length ratios and critical point angles. Using the RET-TORT dataset, they achieved high Spearman correlations for both arteries and veins but acknowledged limitations in generalizability due to resampling dependencies. Similarly, Zhang & Deng (2024) highlighted the integration of functional retinal biomarkers, including vessel tortuosity and perfusion dynamics, as potential early predictors of diabetic retinopathy progression. Their study concluded the diagnostic value of vessel shape descriptors in conjunction with structural biomarkers.

Hervella et al. (2024) leveraged explainable artificial intelligence by combining deep learning with anatomical weighting factors (*e.g*., vessel caliber, artery-vein (AV) type, and distance to optic disc). Their method, trained on multiple datasets including RITE, DRIVE, and the Indian Diabetic Retinopathy Image Dataset (IDRiD), achieved $AUC = 95.21\%$ and was shown to be robust, but it relied heavily on annotated ground truths and anatomical precision. Alternative approaches include the use of eccentricity-based metrics (De Zoysa et al., 2018), curvature estimation filters such as U-COSFIRE (Ramachandran et al., 2020), and differential geometry (Manjunatha, Koti & Sheshadri, 2019). While these methods demonstrate high accuracy in distinguishing tortuosity severity, many struggle with generalization across datasets or did not attempt to separate artery and vein structures. Fully automated pipelines, such as those by Khanal & Estrada (2023) focus on vessel topology extraction and segment-level tortuosity assessment across datasets like DRIVE, HRF, and LES-AV. Their approach shows state-of-the-art performance but may lack transparency for clinical interpretation.

A recurring limitation in much of the literature is the inconsistent treatment of artery and vein tortuosity. Several studies either ignore this distinction or apply it only to subsets of data (*e.g*., RET-TORT), potentially limiting diagnostic applicability. Furthermore, field of view (FOV) and image resolution differences are seldom accounted for, which can affect tortuosity readings due to peripheral vessel inclusion. Table 2 presents a consolidated summary of the studies reviewed, highlighting the diversity in methods, datasets, FOVs, and whether artery-vein separation was performed.

**Table 2:** Comparative summary of retinal vascular tortuosity studies. Methods, datasets, and artery/vein differentiation.

Study/Method	Tortuosity method	Dataset used	Key findings/Limitations	Artery/Vein separated
De Zoysa et al. (2018)	Eccentricity-based Tortuosity Index (ETI)	Local dataset	Significantly differentiates disease stages; robust against elongation/dilation; lacks vessel-type separation	No
Chetia & Nirmala (2019)	Image processing-based white pixel analysis	DRIVE, HRF, RET-TORT, Local	Matches expert grades; sensitive to segmentation quality	RET-TORT: Yes Others: No
Manjunatha, Koti & Sheshadri (2019)	Oscillating Pendulum-Based Algorithm (OPBA)	DRIVE, FIRE, Local	High accuracy in tortuosity and width; affected by slope/angle variations	No
Ramos et al. (2018)	Multiple classical metrics (Hart, Grisan, Trucco, Onkaew)	Local diabetic dataset (60 images)	Best performance from Grisan; expert agreement variability	No
Chen et al. (2020)	Trainable COSFIRE filter and curvature quantification	DRIVE, IOSTAR, Private	High adaptability; effective on nerves and vessels; sensitive to segmentation artifacts	No
Ramachandran et al. (2020)	U-COSFIRE filters with high-curvature point detection	KIDROP	High sensitivity/specificity for ROP; interpretable; some false positives from image artifacts	No
Ramos et al. (2020)	Mathematical metric with anatomical context	Local dataset (200 images)	Increased accuracy with anatomical data; validated with expert scoring	Yes
Wang et al. (2021)	Subdivision-based curvature learning model	RET-TORT, Clinical diabetic dataset	Excellent prediction of DR; high correlation with experts; diabetic-only validation	Yes
Badawi et al. (2023)	Three-level tortuosity measurement using curvature and slope-based descriptors (T1, T2, T3), including pixel tracing from OD to vessel endpoints	DRIVE, STARE, CHASEDB	Retinal vessel type has no statistical significance in the tortuosity calculation results.	Yes
Chuang & Handayani (2023)	Combined parameter of curvature ratios and angular points	RET-TORT (Padova)	Strong expert correlation; limited generalization beyond RET-TORT dataset	Yes
Khanal & Estrada (2023)	Full topology-based vessel analysis using Dijkstra smoothing	DRIVE, HRF, LES-AV, INSPIRE-AVR	Accurate AV classification and tortuosity; full vasculature analyzed	Yes
Giesser et al. (2024)	Vascular curvature index (VCI), arc-over-chord, distance, angle-based, tortuosity density, squared curvature	44 healthy subjects	VCI showed the highest retest reliability among all metrics; limited to healthy subjects, no validation for diseased cases.	Yes
Hervella et al. (2024)	Curvature-based, anatomical weighting, explainable deep learning	Custom, RITE, DRIVE, IDRiD	Human-level accuracy; strong anatomical grounding; relies on annotated ground truth	Yes
Zhang & Deng (2024)	Vascular geometry-based features evaluated through both traditional image processing and machine learning techniques	Fundus photography, OCT angiography	Tortuosity increases before clinical signs of diabetic retinopathy become visible, making it a potential early biomarker; acknowledges that AV differentiation may be important	No

While these advancements have improved measurement accuracy and interpretation, many still suffer from a lack of reproducibility, artery-vein specificity, adaptability across datasets, and scalability for broader clinical deployment. Moreover, limited attention has been given to the influence of FOV and optic disc orientation on tortuosity values, particularly when comparing healthy and pathological cases across various imaging datasets. To address this gap, this study proposes a reproducible and scalable framework that utilizes semi-automatic pixel-level vessel path tracing using the breadth-first search (BFS) algorithm which is chosen for its efficiency in handling complex and branching vessel structures while ensuring complete path reconstruction from the optic disc to peripheral endpoints.

Unlike many prior studies, this research emphasizes both reproducibility and clinical relevance by using only publicly available datasets (DRIVE, HRF, LES-AV), ensuring transparency and validation across multiple imaging settings. Moreover, while most existing works either lack statistical analysis or focus solely on healthy eyes, this study applies comprehensive statistical methods (*e.g*., normality tests, effect size, correlation analysis) to both healthy and diseased cases (diabetic retinopathy and glaucoma).

## Research method

**Figure 1:**
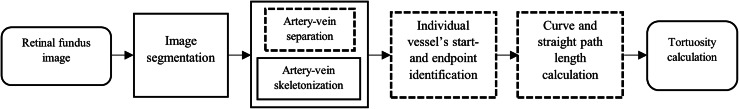
Block diagram of the proposed methodology for tortuosity calculation. The blocks with dashed outlines indicate components newly developed or customized in this study, including artery-vein separation, artery-vein skeletonization, start-endpoint identification, and curve/straight path length calculation.

The proposed methodology builds upon standard retinal image processing steps by introducing several new modules, highlighted with dashed outlines as illustrated by Fig. 1. These include a custom artery-vein separation strategy using red, green, blue (RGB) ground truth overlays, skeletonization of segmented vessels, precise start and endpoint identification using optic disc intersection, and a pixel-level calculation of both curve and straight path lengths based on BFS tracing. Together, these contributions support a reproducible and vessel-specific tortuosity assessment framework. The block diagram components are explained in the following sections.

### Retinal fundus image

Three publicly available databases were used in this study: Digital Retinal Images for Vessel Extraction (DRIVE), High-Resolution Fundus (HRF), and Labelled Eye fundus Segmentation—Artery Vein (LES-AV) (Hu et al., 2021). The DRIVE database consists of 20 colour retinal fundus images of normal eyes, each with a resolution of 584 × 565 pixels with a field of view (FOV) of 45° (Saad, 2025). The HRF database contains 45 images equally divided among three categories, namely, healthy subjects, patients with diabetic retinopathy, and patients with glaucoma. Images were captured with an FOV of 45° and a pixel resolution of 3,304 × 2,336 (Odstrcilik et al., 2013). Meanwhile, the LES-AV database comprises 22 retinal fundus images, including 21 normal images and one image with glaucoma. Of these, 21 images were captured with a 30° FOV at a resolution of 1,444 × 1,620 pixels, while the glaucoma image was acquired with a 45° FOV and a resolution of 1,958 × 2,196 pixels (Orlando et al., 2018). To distinguish arteries and veins in the retinal fundus images, we utilized the RGB ground truth maps provided by Hu et al. (2021), in which arteries, veins, and overlapping vessels are annotated in red, blue, and green, respectively. These RGB ground truth maps are available at https://github.com/yiyg510/VC-Net. The annotations served as the basis for separating the vessel types in our analysis pipeline.

### Image segmentation

**Figure 2:**
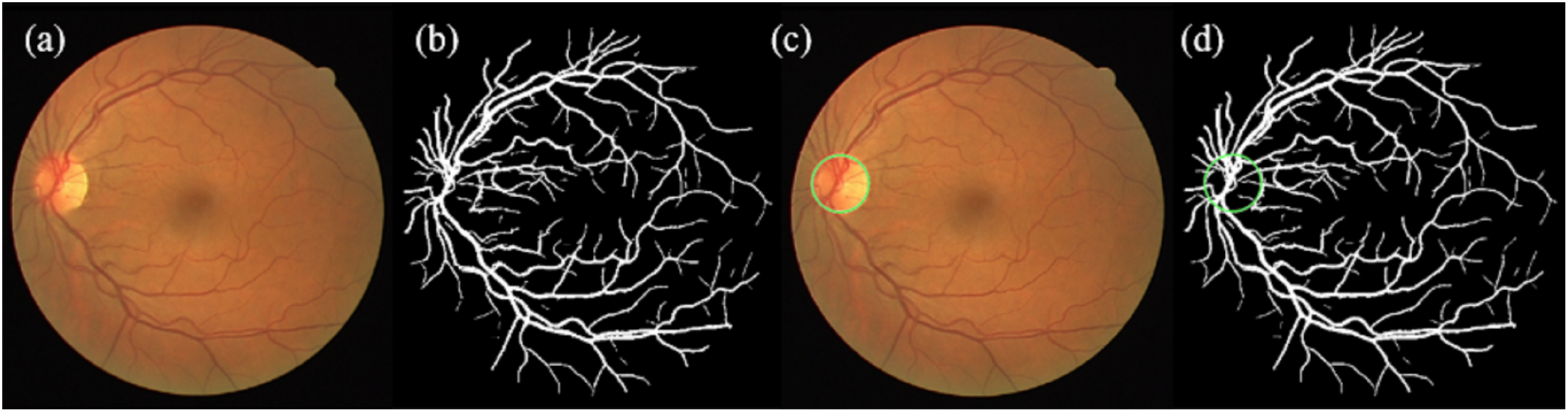
Illustration of normalization, resizing, segmentation, and OD detection steps. (A) Enhanced original image, (b) segmented image, (C) enhanced original image with detected OD, (D) segmented image with detected OD (the OD is indicated in green circle).

Prior to tortuosity analysis, a series of pre-processing steps were applied to each retinal fundus image, based on the vessel segmentation framework proposed by Ali et al. (2021). These steps encompassed normalization, resizing, segmentation, and optic disc (OD) detection. An overview of the process is illustrated in Fig. 2.

During the normalization phase, each image was converted to double precision and normalized to $\left[ {0,\; 1} \right]$ based on Eq. (1) to a predefined intensity range to ensure consistency across datasets.

(1) $${I_{norm}}\left( {x, y} \right) = \displaystyle{{I\left( {x, y} \right)} \over {255}}$$

Images exceeding 1,000 pixels were resized using a fixed resizing factor, $r = 0.5$ to ensure computational efficiency. The pixel-per-degree ratio, $ppd$ was then calculated based on the formula in Eq. (2):

(2) $$ppd = \displaystyle{{{H_{image}}} \over {FOV}}$$

where ${H_{image}}$ is the image height and $FOV$ is field of view. Images with a $ppd > 50$ were further rescaled by 50% to standardize the spatial resolution across datasets.

Various vessel segmentation techniques have been proposed in the literature, including matched filtering (Almotiri, Elleithy & Elleithy, 2018), morphological processing (Fraz et al., 2012), supervised classification methods such as support vector machines and random forests (Strisciuglio et al., 2016), and deep learning-based convolutional neural networks (CNNs) (Oliveira, Pereira & Silva, 2018). Although these methods can achieve high segmentation accuracy, many require high amount of training datasets, or introduce over-segmentation in fine vessels. Meanwhile, Ali, Zaki & Hussain (2019) proposed to integrate the B-COSFIRE filter with adaptive thresholding (hybrid method) to deliver high-quality vessel delineation without extensive training data. The method enables robust detection of vessels of varying widths and orientations while effectively suppressing background noise.

In this study, the vessel segmentation was performed based on the hybrid method. The original image was converted into LAB color space, and the ‘b’ channel was extracted and normalized for vessel enhancement. The Iterative Self-Organizing Data Analysis Technique (ISODATA) (Ball & Hall, 1965) thresholding was then applied to generate a binary vessel mask based on formula in Eq. (3):

(3) $$T = Isodata\left( {{I_b}} \right),\quad M = 1 - \left( {{I_b} < T} \right)$$

where ${I_b}$ the normalized ‘b’ channel. The ISODATA thresholding technique as proposed by Ali, Zaki & Hussain (2019) was adapted due to its proven capability in enhancing vessel detection performance, particularly in capturing fine and low-contrast vessels that are often missed by traditional methods. This thresholding technique offers an unsupervised and adaptive approach that does not rely on pre-labelled data, making it robust across different datasets and imaging conditions. This characteristic is critical especially for tortuosity measurement, as accurate delineation of thin peripheral and branching vessels ensures that curvature is preserved during skeletonization, avoiding underestimation of vessel twistiness. By minimizing missed detections and maintaining vessel continuity, ISODATA supports reliable pixel-level path tracing, which is essential for precise tortuosity computation.

Morphological operations were subsequently employed to remove noise and refine the vascular structure. To enhance detection of both small and large vessels, a custom enhancement technique utilizing BCOSFIRE and Frangi filters was applied. This improved vessel continuity and increased vessel-background contrast.

OD detection was carried out to establish a reference point for subsequent vessel tracing. Morphological preprocessing, adaptive histogram equalization, and Hough circle detection were used on the segmented image to localize the OD. The detection process included scaling factors to account for image resolution differences and validation criteria to ensure accurate localization within the visible vascular area. The OD center and radius were then recorded and rescaled to match the original image dimensions. Visual validation was conducted by overlaying detected OD boundaries on both the original and segmented images by applying Eq. (4):

(4) $$pp{d_{OD}} = \displaystyle{{{R_{OD}}\cdot S} \over {FOV}}$$

where ${R_{OD}}$ is the radius of the OD (in pixel), $S$ is the resizing factor to restore the OD radius back to the original image scale. To further improve vessel detection particularly for small and disconnected retinal blood vessels, an enhancement step was implemented using RGB-labeled ground truth images (Hu et al., 2021). The initial segmented image was overlaid with binary artery and vein masks extracted from RGB ground truth labels. Arteries (red channel) and veins (blue channel) were added back into the map to restore continuity. The RGB image was converted to hue, saturation, value (HSV) color space and classified using hue $\left( H \right)$, saturation $\left( S \right)$, and value $\left( V \right)$ thresholds using Eqs. (5)–(7), respectively:

(5) $${M_{artery\left( {red} \right)}}\left( {x,\; y} \right) = \left\{ {\matrix{ 1\hfill & {if\; \left( {0 \le H \le 0.1 \vee 0.9 \le H \le 1} \right) \wedge S > 0.4 \wedge V > 0.3}\hfill\cr 0\hfill & {otherwise}\hfill\cr} } \right.$$

(6) $${M_{vein\left( {blue} \right)}}\left( {x,\; y} \right) = \left\{ {\matrix{1 \hfill & {if\; 0.55 \le H \le 0.75 \wedge S > 0.4 \wedge V > 0.3}\hfill\cr 0\hfill & {otherwise}\hfill\cr} } \right.$$

(7) $${M_{overlap\left( {green} \right)}}\left( {x,\; y} \right) = \left\{ {\matrix{0\hfill & {if\; 0.2 \le H \le 0.45 \wedge S > 0.3 \wedge V > 0.3}\hfill\cr 0\hfill & {otherwise}\hfill\cr} }. \right.$$

**Figure 3:**
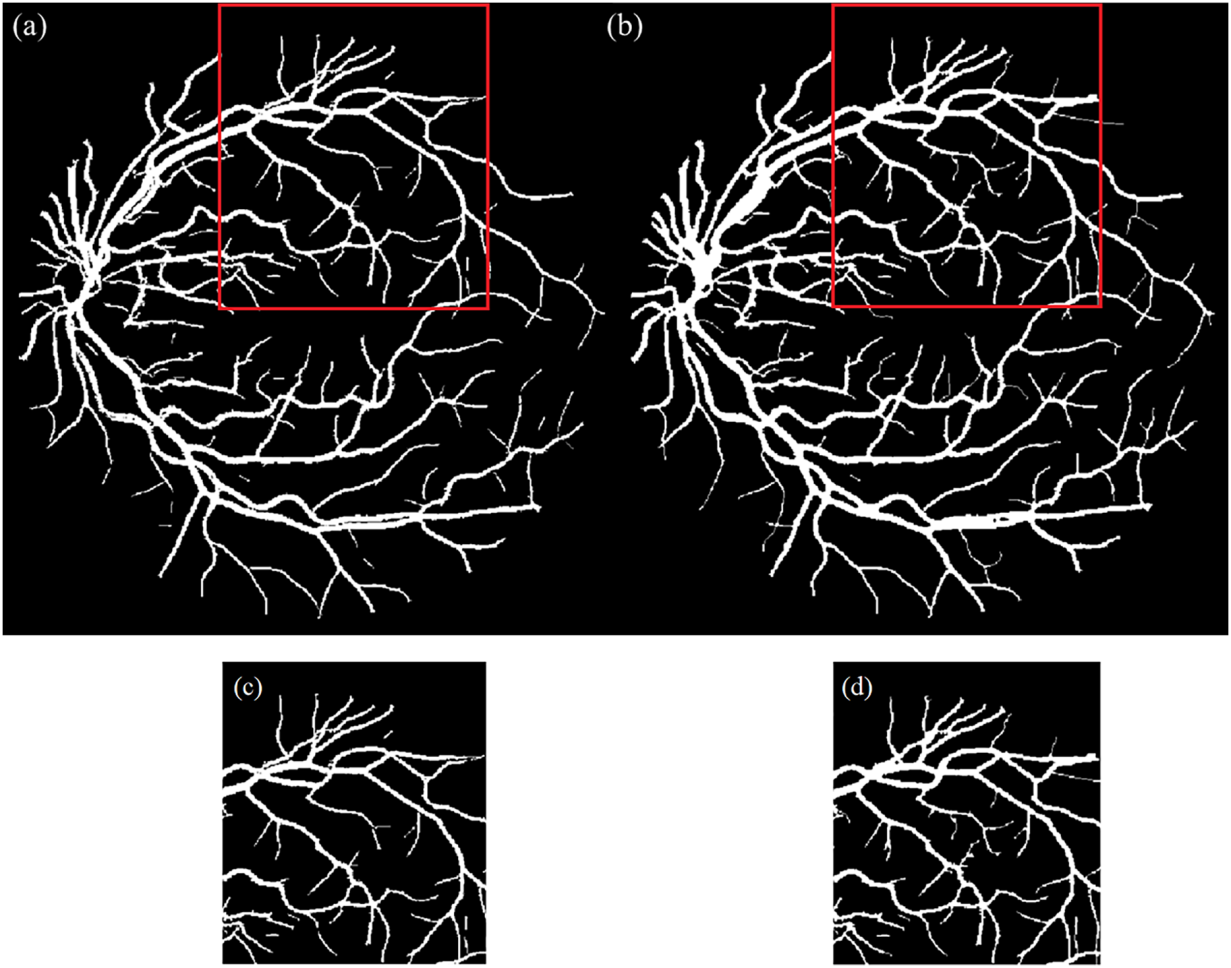
Illustration of segmented image. (A) Before and (B) after the vessel enhancement to restore the vessel continuity and capture small and disconnected vessels; (C) & (D) are the enlarged red boxes in (A) & (B), respectively.

The final vessel map was constructed by combining all binary regions. Next, to improve continuity and eliminate small gaps especially in thin or bifurcated vessel regions a morphological closing operation was applied using a disk-shaped structuring element. Figure 3 illustrates the vessel map before and after enhancement, showing improvements in vessel continuity and visibility of fine vascular structures.

### Artery-vein separation and skeletonization

To enable artery-vein-specific tortuosity analysis, the enhanced binary vessel segmentation image was overlaid with an RGB ground truth annotation map that classified arteries in red, veins in blue, and overlapping vessels in green, as defined by Hu et al. (2021). This overlay enabled the separation of the vascular structure into distinct artery and vein masks while preserving continuity in regions of overlap. Prior to processing, the RGB ground truth image was resized to match the resolution of the segmented binary image using nearest-neighbor interpolation. This ensured accurate pixel-wise alignment during mask generation.

The RGB image was then converted to HSV color space to facilitate robust color-based classification. Color thresholds were applied to extract binary masks for arteries, veins, and overlapping vessels based on predefined hue, saturation, and brightness values. The artery and vein masks were constructed by intersecting the classified color regions with the segmented vessel map. To maintain full vascular connectivity, pixels corresponding to overlapping vessels were included in both artery and vein masks. Morphological refinement was performed using a disk-shaped structuring element to eliminate minor discontinuities and enhance structural consistency. This operation ensured that the separated vessel maps remained topologically intact for further analysis.

Arteries and veins exhibit different wall composition and baseline geometry, leading to vessel-specific tortuosity characteristics and disease effects (Alam, Le & Yao, 2021; Duh, Sun & Stitt, 2017). Without separation, it can obscure these differences and biased outcomes. Maintaining separate artery and vein maps supports vessel-specific statistics and artery-vein contrasts within eyes, improves BFS path tracing at crossings by reducing class ambiguity, and enhances reproducibility when comparing datasets with different FOVs and image orientations.

Skeletonization was then applied independently to the artery and vein maps to reduce each vessel to a one-pixel-wide centerline while preserving path topology. This step both simplifies subsequent computations and provides stable centerline coordinates (Nasiruddin et al., 2022) for width/intensity sampling and path tracing. The skeletonization process involved grayscale conversion, binarization, and iterative thinning, yielding simplified yet structurally representative maps suitable for tracing and measurement. Mathematically, the skeleton $S\left( I \right)$ of a binary vessel image III can be expressed as Eq. (8):

(8) $$S\left( I \right) = \mathop \bigcup \limits_{k = 0}^K \left( {I\ominus kB} \right)\backslash \left( {\left( {I\ominus kB} \right) \circ B} \right)$$

where $B\;$is the structuring element (disk), $\ominus $ denotes erosion and $\circ$ denotes opening (Kresch & Malah, 1998; Maragos & Schafer, 1986).

**Figure 4:**
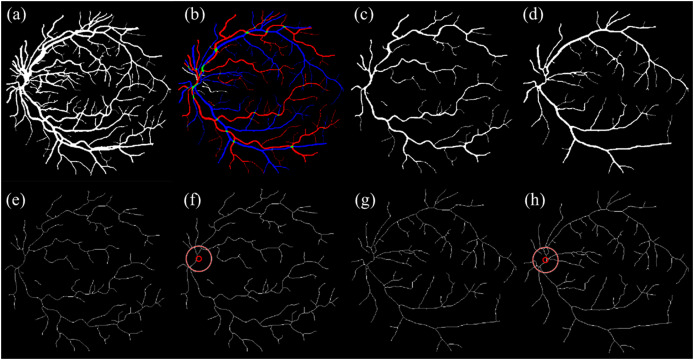
Illustration of artery-vein separation. (A) Enhanced segmented image and (B) RGB ground truth. (C) Separated artery. (D) Separated vein. (E) Skeletonized artery. (F) Skeletonized artery with OD. (G) Skeletonized vein. (H) Skeletonized vein with OD.

The OD location previously detected during segmentation was then overlaid as a circular marker on each skeletonized image. This ensured that vessel path tracing was anchored to a consistent anatomical origin. Figure 4 illustrates the key stages of artery-vein separation and skeletonization.

The pseudocode for the artery-vein separation and skeletonization is shown below:

Data: S – binary segmented vessel map;

       G – RGB ground-truth labels (red = artery, blue = vein, green= overlap);

       OD – optic disc center & radius (optional).

Result: A_skel, V_skel; A_skel_OD, V_skel_OD.

initialization;

resize G to size(S);

convert G to HSV → (H, S, V);

M_Artery ← threshold red on (H, S, V);

M_Vein ← threshold blue on (H, S, V);

M_Overlap ← threshold green on (H, S, V);

Artery ← S AND (M_Artery OR M_Overlap);

Vein ← S AND (M_Vein OR M_Overlap);

Artery ← morphological closing(A, disk(2));

Vein ← morphological closing(V, disk(2));

Artery_skel ← iterative thinning(Artery);

Vein_skel ← iterative thinning(Vein);

if OD is available then

     Artery_skel_OD ← overlay OD on Artery_skel;

     Vein_skel_OD ← overlay OD on Vein_skel;

end

save Artery_skel, Vein_skel, Artery_skel_OD, Vein_skel_OD;

end

### Individual vessel’s start- and endpoint identification

Accurate tortuosity computation relies on identifying clinically meaningful vessel paths, beginning at the OD and extending to terminal vessel endpoints. This section describes the methodology used to detect start and end points of each individual vessel segment. All white pixels from skeletonized retinal vasculature images, representing the vessel centerlines, are extracted by identifying their coordinates. All vessel pixels are extracted as coordinate pairs as calculated based on Eq. (9):

(9) $$P = \left\{ {\left( {{x_i},\; {y_i}} \right)|\; {I_{skel}}} \right.\left( {{x_i},\; {y_i}} \right) = 1$$

where ${I_{skel}} \in {\left\{ {0,\; 1} \right\}^{H \times W}}$ is binary skeleton image, where vessel centerline pixels are white.

To determine suitable start points, previously computed OD coordinates were overlaid onto the skeletonized image to ensure spatial alignment. The Euclidean distance between each vessel pixel and the OD center was computed using Eq. (10):

(10) $${d_i} = \sqrt {{{\left( {{x_i} - {x_{OD}}} \right)}^2} + \; {{\left( {{y_i} - {y_{OD}}} \right)}^2}}$$

where ${\left( {{x_i} - {x_{OD}}} \right)^2}$ is OD center coordinates and ${r_{OD}}$, the Euclidean distance from each pixel $\left( {{x_i},{y_i}} \right) \in P$ to the OD center. Pixels satisfying ${r_{OD}} \le {d_i} \le {r_{OD}} + \delta$, where $\delta$ is a small buffer, are considered valid starting points. Vessel pixels lying along or near the OD boundary were considered potential starting points for tracing, as they represent anatomical continuity from the optic nerve.

Next, vessel endpoints defined as the outermost terminal points on the vascular branches were identified. Endpoints are detected using morphological endpoint detection on ${I_{skel}}$. For each endpoint $\left( {{x_e},{y_e}} \right)$, the same Euclidean distance formula is applied. Using the known OD center and radius, a distance threshold was endpoints closer than the OD radius were discarded to ensure that only distal, anatomically relevant segments were retained. This means, any endpoint with $\left( {{d_e} \le {r_{OD}}} \right)$ is excluded to ensure that the endpoint lies outside the OD boundary.

To ensure anatomical continuity, a BFS algorithm was employed from each candidate endpoint $e \in \delta$ to trace a path through the vessel skeleton, ${I_{skel}}$. If the traversal successfully reached any of the previously defined start points, the endpoint was validated as belonging to a complete and traceable vessel path.

The graph $G = \left( {P,\; \varepsilon } \right)$ is modeled as an undirected graph where vessel pixels are nodes and 8-connected neighbors define edges. A path ${\tau _{s,\; e}}$ is considered valid if the value in Eq. (11):

(11) $$\exists \; path\; {\tau _{s,\; e}} = \left\{ {\left( {{x_0},\; {y_0}} \right),\; \left( {{x_1},\; {y_1}} \right), \ldots ..,\left( {{x_n},\; {y_n}} \right)} \right\}$$

where $\left( {{x_0},\; {y_0}} \right) \in S$, and $S$ is the set of valid startpoints.

**Figure 5:**
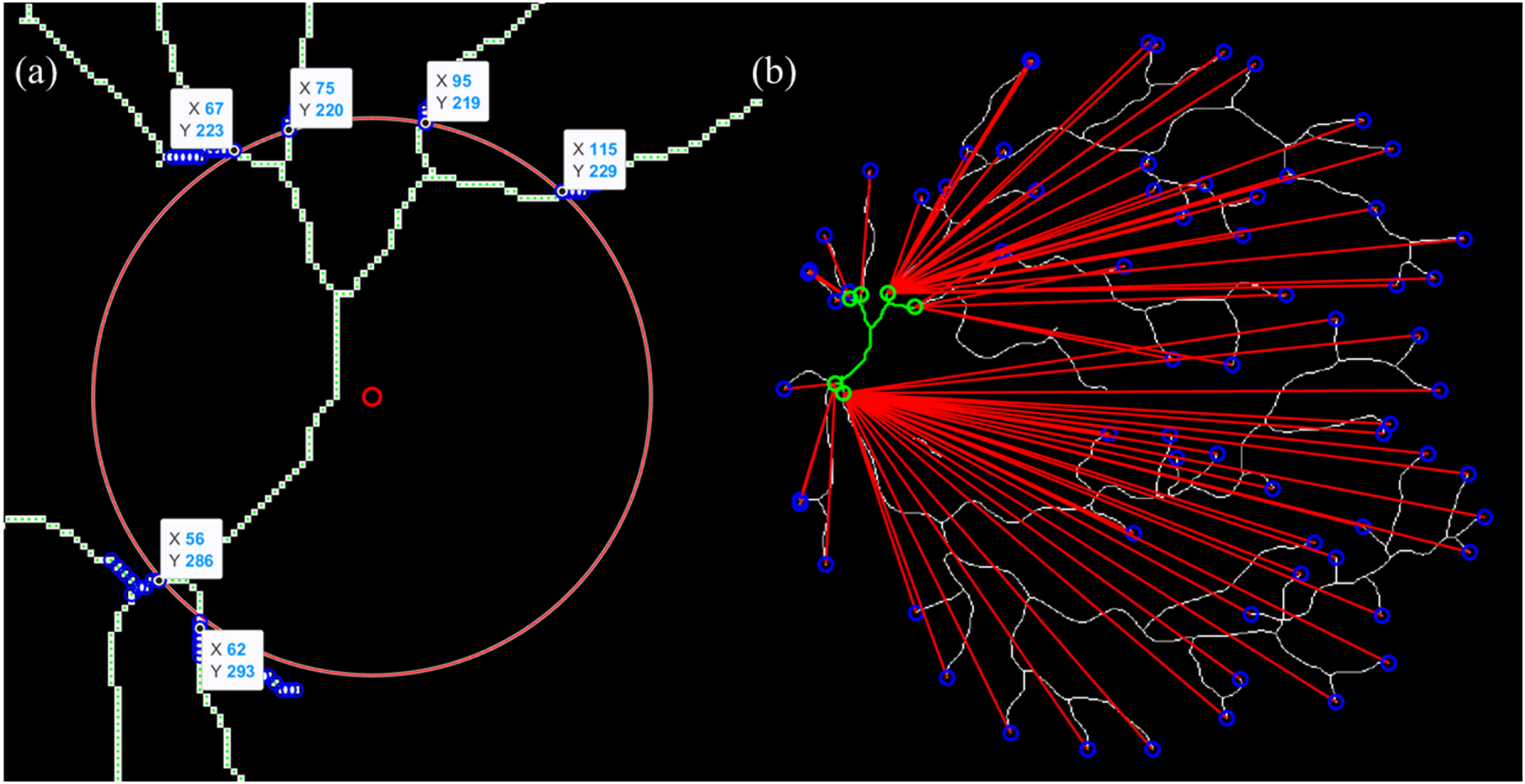
Illustration of the vessel start- and endpoint identification (sampled for artery). (A) The intersection point between vessel and OD is presumed as a startpoint. (B) Endpoint identification for each and vessel tracing from start- to endpoints.

The BFS algorithm was chosen for vessel path tracing due to its efficiency, simplicity, and suitability for traversing binary skeleton maps of retinal vessels like it did in multi-branching and stenoses (Fang et al., 2018; Huang et al., 2018; Li, Dankelman & De Momi, 2021). BFS operates by exploring all neighboring pixels level by level, ensuring that the shortest topological path is found between the OD (startpoint) and vessel endpoints. This property is crucial for arc length calculation in tortuosity, where accurate and complete path tracing is required. Figure 5 illustrates an example of start- and endpoint identification for an arterial segment.

The pseudocode for the vessel’s start- and endpoint identification is shown as below:

*Data: SKEL – skeletonized vessel map (binary);*

*       OD – optic disc center (x_od, y_od) and radius r_od;*

*      STARTS_manual (optional) – user-specified start points.*

*Result: PAIRS – set of (start, end) vessel pairs outside OD.*

*initialization;*

*ensure SKEL is binary;*

*load OD; build OD_mask = {p : dist(p,OD) ≤ r_od};*

*W ← coordinates of white pixels in SKEL;*

*δ ← 5;                           // margin beyond OD*

*CAND_STARTS ← { w ∈ W : r_od < dist(w,OD) ≤ r_od+δ };*

*if STARTS_manual provided then*

*      STARTS ← STARTS_manual;*

*else*

*     STARTS ← CAND_STARTS;*

*end*

*ENDS ← endpoints(SKEL);*

*while ENDS not empty do*

*    e ← pop(ENDS);*

*    if dist(e,OD) > r_od then*

*        p ← BFS on SKEL from e to any s ∈ STARTS avoiding OD_mask;*

*       if p exists then*

*             PAIRS ← PAIRS* ∪ *{(start(p), e)};*

*        end*

*    end*

*end*

*export PAIRS to table (Start (x,y), End (x,y));*

*end*

### Curve and straight path length calculation

The arc length is derived from the vessel’s skeleton path, while the straight-line length represents the shortest geometric distance between the start- and endpoint. Each vessel path defined by the validated start and endpoint pair $\left( {S,\; e} \right)$ is used to compute tortuosity based on its arc and straight-line lengths. The arc length is calculated by performing BFS traversal from $S$ to $e$, tracing the entire connected path through the graph $G$. The total arc length is defined as Eq. (12):

(12) $${L_{curve}} = \vert {{\tau _{s,e}}} \vert$$

where ${L_{curve}} = \left|\; {\cdot}\; \right|$ denotes the number of pixels along the traced vessel path. The straight-line path length between each start- and endpoint pair was calculated using the Euclidean distance formula as stated in Eq. (13):

(13) $${L_{straight}} = \sqrt {{{\left( {{x_s} - {x_e}} \right)}^2} - {{\left( {{y_s} - {y_e}} \right)}^2}}.$$

where $\left( {{x_s},\; {y_s}} \right)$ and $\left( {{x_e},\; {y_e}} \right)$ represent the coordinates of the start- and endpoint, respectively. This distance reflects the minimum geometric path and is used to normalize the arc length, enabling standardized tortuosity comparison across vessel segments.

**Figure 6:**
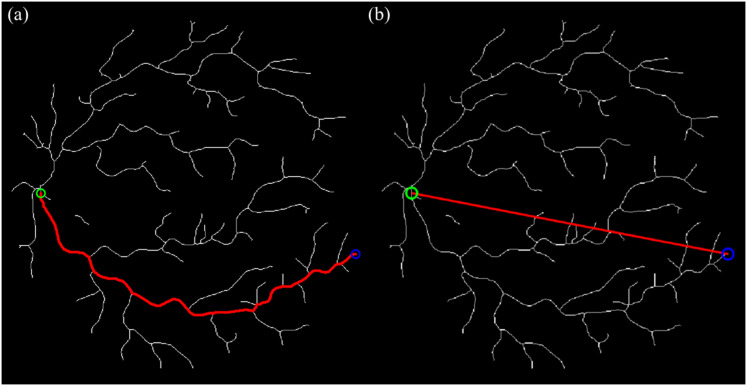
The curve & straight path length calculation using BFS algorithm and Euclidean distance technique (sampled for artery). (A) Tracing for curve length from start- to endpoint. (B) Tracing for straight length from start- to endpoint.

We use the Euclidean endpoint distance as the straight-line term because it is a closed-form and parameter-free baseline introduced by Heneghan et al. (2002), Fraz et al. (2015) that depends only on the startpoint and endpoint coordinates, making it easy to implement and replicate across datasets; it also matches the standard definition of tortuosity widely used in retinal studies (arc length divided by Euclidean distance), ensuring comparability with prior work. The curve and straight-line tracing processes are illustrated in Fig. 6.

The pseudocode for the curve and straight path length calculation is shown as below:

*Data: SKEL – skeletonized vessel map (binary);*

*       PAIRS_XLS – spreadsheet with pairs: Startpoint (x, y), Endpoint (x, y).*

*Result: CURVE_LEN, STRAIGHT_LEN per pair; Excel tables (E2, E3).*

*initialization;*

*ensure SKEL is binary;*

*read PAIRS_XLS → P = {(s_i, e_i)}; // parse "(x, y)" strings to integers*

*for each (s, e) in P do*

*    // Curve (arc) length via BFS on 8-connected skeleton*

*    queue ← [s]; visited ← {s}; prev ← ∅ found ← false;*

*    while queue not empty do*

*        u ← pop_front(queue);*

*        if u == e then found ← true; break; end*

*        for v in N8(u) do*

*            if v in-bounds and SKEL(v)=1 and v ∉ visited then*

*                 visited ← visited* ∪ *{v}; prev[v] ← u; push_back(queue, v);*

*           end*

*      end*

*  end*

*  if found then*

*       path ← backtrack(prev, e → s);*

*      CURVE_LEN[i] ← |path|; // pixels along skeleton*

*  else*

*      CURVE_LEN[i] ← NaN;*

*  end*

*   // Straight-line length (Euclidean)*

*   STRAIGHT_LEN[i] ← round(sqrt((e.x−s.x)^2 + (e.y−s.y)^2));*

*end*

### Tortuosity results

We adopt the standard definition of tortuosity as the ratio of curve (arc) length to the straight-line (Euclidean) distance between endpoints, as utilized in Mustafar et al. (2023) study. The tortuosity, $T$ for a vessel segment is calculated as the ratio of the arc length to the straight-line length, given in Eq. (14):

(14) $$T = \displaystyle{{{L_{curve}}} \over {{L_{straight}}}}.$$

This ratio reflects the degree of “twistiness” or curvature in the vessel. A perfectly straight vessel would yield a tortuosity value of 1, whereas increasingly curved vessels would result in higher values. The resulting tortuosity values were statistically analyzed to evaluate their behavior across datasets (DRIVE, HRF, LES-AV), between vessel types (artery *vs*. vein), and between health conditions (healthy *vs*. diabetic retinopathy *vs*. glaucoma).

## Results

This section reports the findings from the analyses conducted to evaluate retinal vessel tortuosity across different datasets, vessel types, disease conditions and imaging settings.

### Artery-vein tortuosity patterns in healthy eyes under varying field of view (FOV) settings

Table 3 summarizes the descriptive statistics of artery and vein tortuosity in healthy eyes across three datasets: DRIVE, HRF, and LES-AV.

**Table 3:** Results of the artery *vs*. vein tortuosity behavior of healthy eyes in all datasets.

Dataset		FOV	Total	Mean tortuosity	Shapiro-Wilk *p*-value	Pearson correlation	Pearson *p*-value	Linear regression	Cohen’s
					$p$	$r$	$p$	${R^2}$	$d$
DRIVE: 20 images	Artery	45°	1,211	1.108	0.2642	0.5608	0.0101	0.3145	−1.5859
	Vein		1,577	1.22	0.0703				
HRF: 15 images	Artery	45°	1,595	1.1167	0.1322	0.0998	0.7235	0.01	−2.4707
	Vein		1,758	1.2113	0.4510				
LES−AV: 11 images	Artery	30°	326	1.02	0.9446	0.1994	0.5566	0.0398	−1.0112
	Vein		382	1.0709	0.9421				

Referring to Table 3, the statistics indicate that veins from DRIVE dataset (mean $= 1.22$) exhibit higher tortuosity than arteries (mean $= 1.108$), with greater variability and heavier distribution tails. Both artery and vein tortuosity follow a normal distribution (Shapiro-Wilk $p > 0.05$), supporting the use of parametric tests. The Pearson correlation ( $r = 0.5608,\; p = 0.01$) shows a moderate and statistically significant relationship, suggesting that increases in arterial tortuosity are moderately associated with increases in venous tortuosity. However, the linear regression model explains only $31.45\%$ of vein tortuosity variance ( ${R^2} = 0.3145$), indicating limited predictive capability. The Cohen’s $d$ of $- 1.5859$ confirms a large effect size, which highlights a significant difference between artery and vein tortuosity. These findings demonstrate that veins are significantly more tortuous than arteries in the DRIVE dataset. While arterial is moderately correlated, arterial tortuosity is not a reliable predictor of venous tortuosity.

The HRF dataset reaffirms findings from the DRIVE dataset. Veins (mean $= 1.2113$) again have higher tortuosity than arteries (mean $= 1.1167$). Distributions are normal (Shapiro-Wilk $\rho > 0.05$), but the Pearson correlation ( $r = 0.0998,\; p = 0.7235$) is weak and not statistically significant. Linear regression further shows poor predictability ( ${R^2} = 0.01$). Nonetheless, the Cohen’s $d$ of $- 2.4707$ reflects an extremely large effect size, indicating substantial differences in tortuosity despite the absence of correlation. These results suggest that while artery and vein tortuosity differ significantly in magnitude, they are not linearly related, pointing to independent mechanisms of structural regulation.

In the LES-AV dataset, veins (mean $= 1.0709$) are again more tortuous than arteries (mean $= 1.02$), although the difference of $0.0509$ is smaller than in DRIVE and HRF. Normality holds for both vessel types (Shapiro-Wilk $p > 0.05$). Pearson correlation ( $r = 0.1994,\; p = 0.5566$) is weak and non-significant, and regression analysis explains only 3.98% of the variance ( ${R^2} = 0.0398$). Still, a large effect size (Cohen’s $d = - 1.0112$) supports a significant difference between arteries and veins. In conclusion, although correlation is weak, the statistically large difference in tortuosity remains consistent with prior datasets, indicating the structural distinction between arteries and veins.

**Figure 7:**
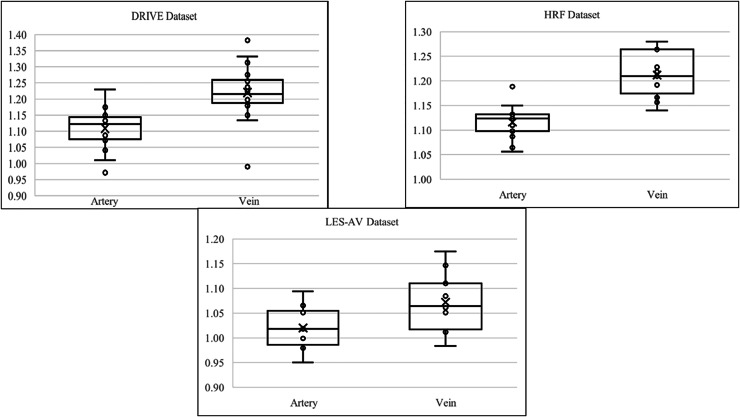
Boxplot comparison of artery and vein tortuosity of healthy eyes in all datasets. Veins consistently show higher tortuosity than arteries across all datasets.

Figure 7 shows the boxplot of artery and vein tortuosity in healthy eyes from the DRIVE, HRF, and LES-AV datasets. Veins are consistently more tortuous than arteries across all datasets, supporting the results in Table 3.

**Figure 8:**
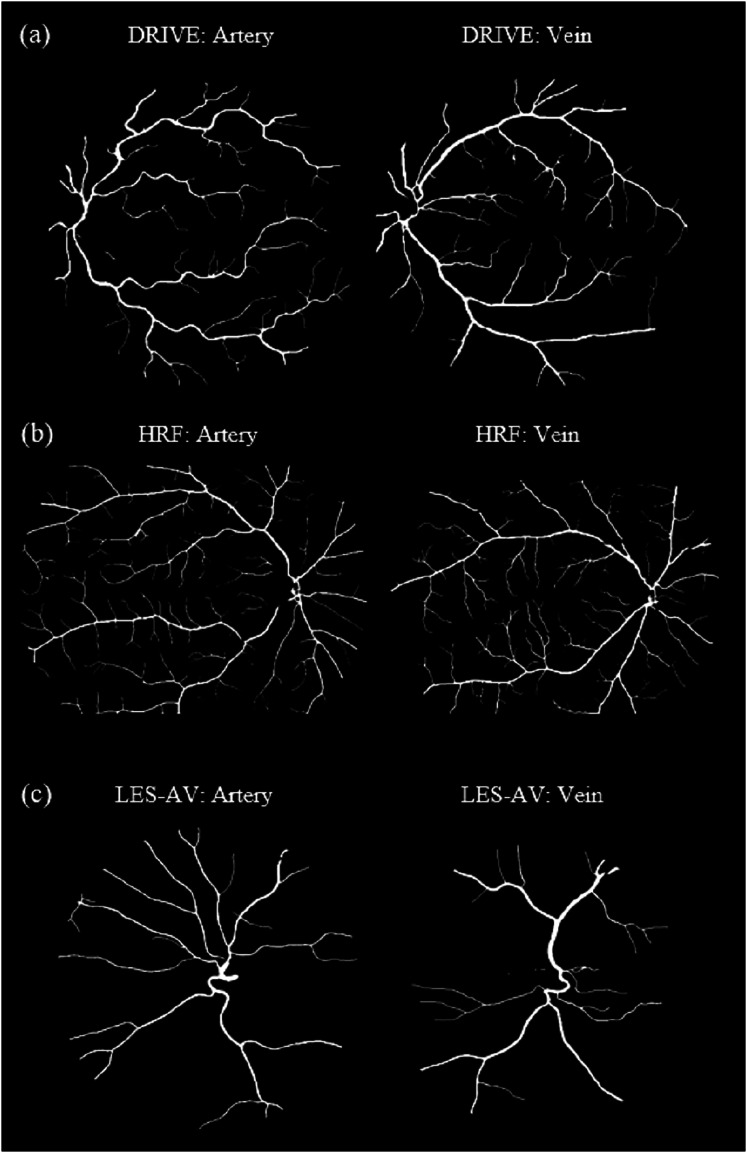
Samples of segmented artery and vein of healthy eyes in all datasets. Veins have obviously higher tortuosity than arteries across all datasets. Meanwhile, the field of view (FOV) significantly influences tortuosity measurements, as a narrower view limits the visibility of peripheral vessels, resulting in artery and vein tortuosity values that appear more similar. (A) DRIVE sample for artery and vein (FOV 45°). (B) HRF sample for artery and vein (FOV 45°). (C) LES-AV sample for artery and vein (FOV 30°).

Figure 8 shows samples of segmented artery and vein of healthy eyes for in all datasets.

### Artery-vein tortuosity patterns in healthy and diseased eyes (HRF dataset)

Table 4 summarizes the results of the experimental analysis, which was conducted from three groups: Healthy eyes, glaucoma cases and diabetic retinopathy (DR) cases.

**Table 4:** Results of the tortuosity of healthy and diseased eyes in HRF dataset.

Dataset		Total	Mean tortuosity	Shapiro-wilk *p*-value	Pearson correlation	Pearson *p*-value	Linear regression	Cohen’s
				$p$	$r$	$p$	${R^2}$	$d$
Healthy: 15 images	Artery	1,595	1.1167	0.1322	0.0998	0.7235	0.01	−2.4707
	Vein	1,758	1.2113	0.4510				
DR: 15 images	Artery	1,134	1.1207	0.8485	−0.3924	0.148	0.154	−2.709
	Vein	1,398	1.2427	0.0622				
Glaucoma: 15 images	Artery	1,537	1.118	0.5695	0.2009	0.4728	0.0404	−1.9775
	Vein	1,716	1.2053	0.6437				

Referring to Table 4, glaucomatous eyes show mean tortuosity of arteries is $1.118$ and $1.2053$ for vein. Distributions are approximately normal, confirmed by the Shapiro-Wilk test (artery $p = 0.5695$; vein $p = 0.6437$). Pearson correlation is weak ( $r = 0.2009,\; p = 0.4728$), and the linear regression yields low predictive power ( ${R^2} = 0.0404$). However, Cohen’s $d = - 1.9775$ indicates a very large difference arteries and veins tortuosity. Similarly, in healthy eyes, the artery mean is $1.1167$ and vein mean is $1.2113$. Correlation remains weak ( $r = 0.0998,\; p = 0.7235$), with low regression ${R^2} = 0.01$, and Cohen’s $d = - 2.4707$ again reflects a large effect size. These findings suggest that both healthy and glaucomatous eyes demonstrate a large tortuosity difference between arteries and veins. Despite these magnitude differences, artery and vein tortuosity values are not significantly correlated.

Meanwhile, in DR cases, the mean tortuosity of arteries is $1.1207$, while veins show a higher mean of $1.2427$. Shapiro-Wilk test confirms normality (artery $p = 0.8485$; vein $p = 0.0622$). A moderate negative Pearson correlation ( $r = - 0.3924,\; p = 0.148$) is observed, though not statistically significant. Linear regression explains $15.40{\rm \% }$ of the variation ( ${R^2} = 0.154$), and Cohen’s $d$ of $- 2.709$ indicates an extremely large effect size. In comparison, healthy arteries and veins have means of $1.1167$ and $1.2113$, respectively, with weak correlation ( $r = 0.0998,\; p = 0.7235$) and ${R^2} = 0.01$. Thus, DR eyes exhibit an even greater tortuosity difference between arteries and veins than healthy eyes. While correlation remains weak, the magnitude of the difference is amplified in the presence of DR.

**Figure 9:**
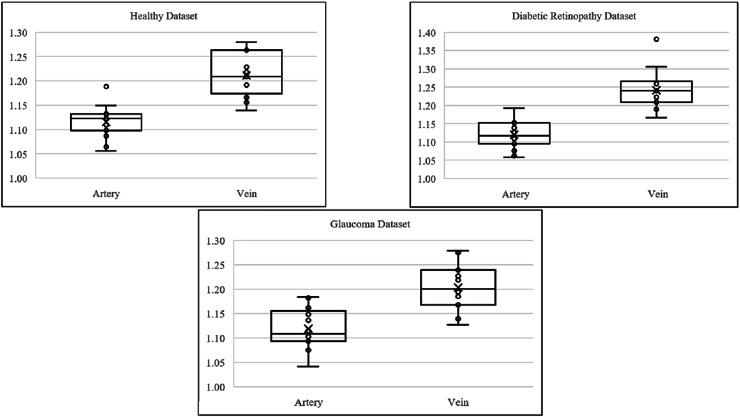
Boxplot comparison of artery and vein tortuosity of healthy and diseased eyes in the HRF dataset. Across all conditions, veins demonstrate consistently higher tortuosity than arteries, with the greatest disparity observed in the diabetic retinopathy group. These visualizations corroborate the quantitative analysis presented in Table 4.

Figure 9 presents boxplots illustrating the distribution of tortuosity values for arteries and veins across three conditions: healthy, DR and glaucoma based on the HRF dataset. In all groups, veins consistently exhibit higher tortuosity than arteries, with the largest difference observed in the DR group. These visual trends reinforce the statistical findings in Table 4, confirming that tortuosity is not only elevated in diseased states but also shows vessel-type specificity (vein).

**Figure 10:**
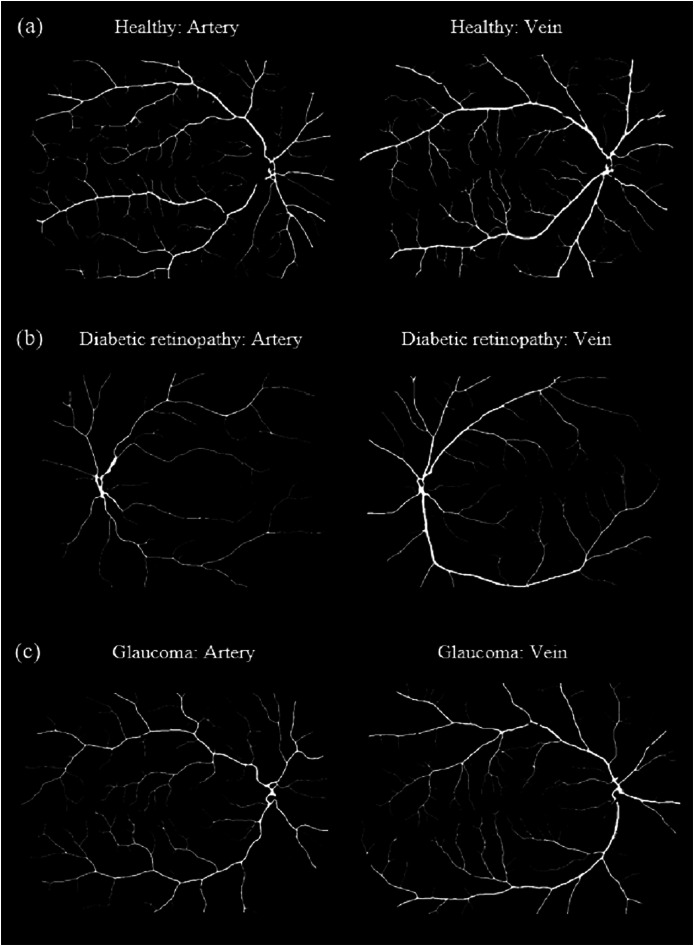
Samples of segmented artery and vein of healthy and diseased eyes in the HRF dataset. Veins consistently exhibit higher tortuosity than arteries, with the largest difference observed in the diabetic retinopathy group. (A) Sample for artery and vein (healthy). (B) Sample for artery and vein (diabetic retinopathy). (C) Sample for artery and vein (glaucoma).

Figure 10 shows samples of segmented artery and vein of healthy and diseased eyes in the HRF dataset.

### Artery-vein tortuosity patterns in healthy and glaucoma eyes (LES-AV dataset)

Table 5 summarizes the results of the experimental analysis, which was conducted from two groups: Healthy eyes and glaucoma cases.

**Table 5:** Results of the tortuosity of healthy and glaucomatous eyes in the LES-AV dataset.

Dataset		Total	Mean tortuosity	Shapiro-wilk $p -$value	Pearson correlation	Spearman correlation	Pearson *p*-value	Linear regression	Cohen’s	Mann–whitney U	Wilcoxon
				$p$	$r$	$r$	$p$	${R^2}$	$d$	*p*	*p*
Healthy: 15 images	Artery	1,595	1.02	0.9446	0.1994	–	0.5566	0.0398	1.0112	–	–
	Vein	1,758	1.0709	0.9421							
Glaucoma: 11 images	Artery	248	0.9791	0.0157	–	−0.6402	0.0338	–	–	0.642	0.5488
	Vein	257	0.9936	0.4394							

In the LES-AV dataset, a comparison between healthy and glaucomatous eyes reveals a reduction in both artery and vein tortuosity among glaucoma cases. Specifically, healthy eyes had a mean tortuosity of $1.02$ for arteries and $1.0709$ for veins, while glaucomatous eyes had lower values of $0.9791$ and $0.9936$, respectively.

Normality testing using the Shapiro-Wilk test showed that both healthy arteries and veins were normally distributed ( $p = 0.9446$ and $0.9421$, respectively). However, glaucoma artery data failed the normality test ( $p = 0.0157$), therefore a non-parametric statistical method is applied for analysis.

In healthy eyes, Pearson correlation revealed a weak and non-significant relationship between artery and vein tortuosity ( $r = 0.1994,\; p = 0.5566,\; {R^2} = 0.0398$). In contrast, glaucomatous eyes demonstrated a moderate negative Spearman correlation ( $r = - 0.6402,\; p = 0.0338$), suggesting that as artery tortuosity decreases, vein tortuosity tends to increase.

Mann–Whitney U ( $p = 0.642$) and Wilcoxon signed-rank ( $p = 0.5488$) tests indicate no statistically significant difference between artery and vein tortuosity in glaucoma cases. This contrasts with the healthy group, where a large effect size (Cohen’s $d = - 1.0112$) highlighted a significant difference.

**Figure 11:**
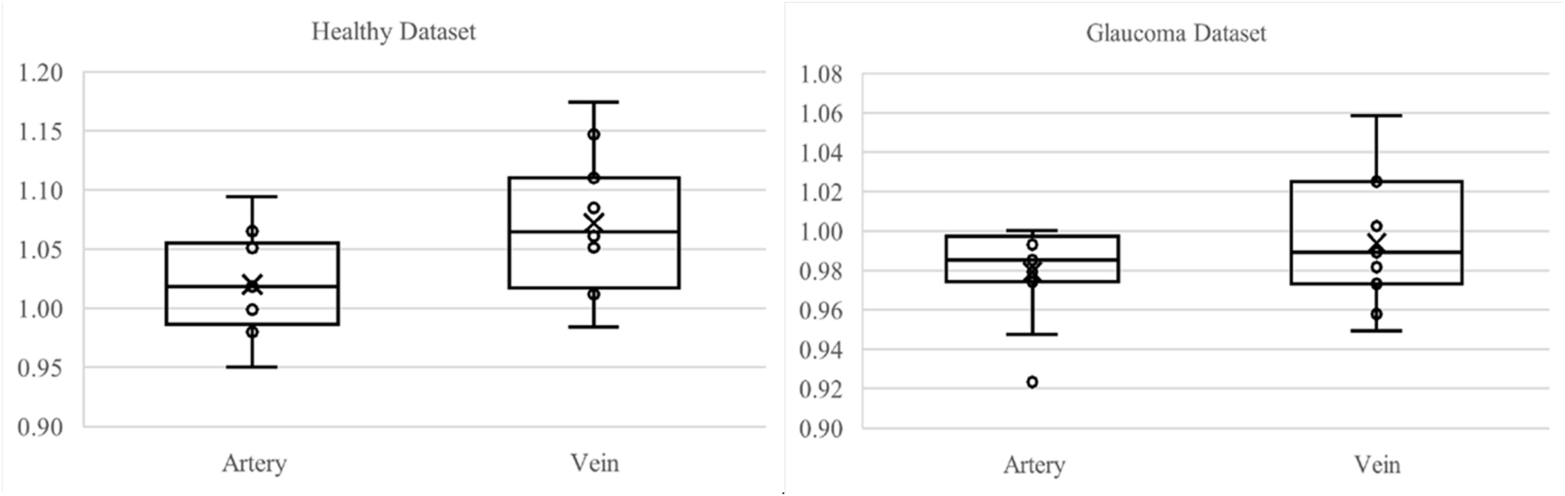
Boxplot comparison of artery and vein tortuosity in healthy and glaucomatous eyes from the LES-AV dataset. Across both groups, veins exhibit greater tortuosity than arteries, consistent with the quantitative analysis summarized in Table 5.

Figure 11 presents boxplots comparing artery and vein tortuosity in healthy and glaucomatous eyes from the LES-AV dataset. In the healthy group, veins show noticeably higher tortuosity than arteries, with a wider interquartile range and slightly higher maximum values, indicating greater variability in venous tortuosity. In glaucomatous eyes, both artery and vein tortuosity values are reduced compared to the healthy group. The distinction between arteries and veins becomes less pronounced, suggesting that glaucoma may attenuate the typical tortuosity difference observed in normal eyes. These visual trends confirm the findings in Table 5, where statistical analyses confirmed lower tortuosity in glaucomatous eyes and a diminished artery-vein difference relative to healthy controls.

**Figure 12:**
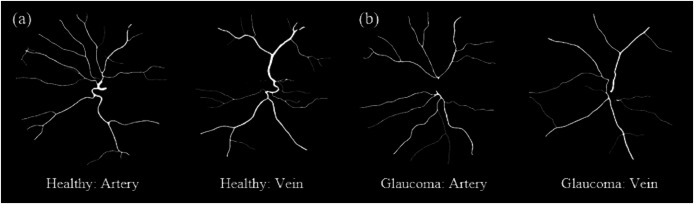
Samples of segmented artery and vein tortuosity of healthy and glaucomatous eyes in the LES-AV dataset. Veins show noticeably higher tortuosity than arteries in healthy group. In glaucomatous eyes, both artery and vein tortuosity values are lower. (A) Sample for artery and vein (healthy). (B) Sample for artery and vein (glaucoma).

Figure 12 shows samples of segmented artery and vein of healthy and glaucomatous eyes in the LES-AV dataset.

### Artery-vein tortuosity patterns in diseased eyes under different field of view (FOV) settings (HRF and LES-AV)

Table 6 concludes the results of the experimental analysis, which was conducted from two datasets, HRF and LES-AV for glaucoma case.

**Table 6:** Results of the tortuosity of glaucomatous eyes in the HRF and LES-AV datasets.

Dataset		FOV	Total	Mean Tortuosity	Shapiro-Wilk *p–*value	Pearson correlation	Spearman correlation	Pearson *p* value	Linear regression	Cohen’s	Mann–Whitney U	Wilcoxon
					*P*	*r*	*r*	*P*	R^2^	*d*	*p*	*p*
HRF: 15 images	Artery	45°	1,537	1.02	0.9446	0.2009	–	0.4728	0.0404	−1.9775	–	–
	Vein		1,716	1.0709	0.9421							
LES-AV: 11 images	Artery	30°	248	0.9791	0.0157	–	−0.6402	0.0338	–	–	0.642	0.5488
	Vein		257	0.9936	0.4394							

This analysis explores whether tortuosity behavior in glaucomatous eyes differs between two datasets with different imaging characteristics: HRF (field of view: $45^\circ$) *vs*. LES-AV (field of view: 30°). Despite sharing the same disease classification, variations in field of view, image resolution, and labeling standards may contribute to differences in measured tortuosity.

In the HRF dataset, arteries exhibit a mean tortuosity of $1.02$ and veins 1.0709. The tortuosity distributions in both vessel types were found to be approximately normal (Shapiro-Wilk $p > 0.05$), supporting the use of parametric tests. Pearson correlation between arteries and veins was weak ( $r = 0.2009,\; p = 0.4728$), and regression analysis confirmed a low predictive value ( ${R^2} = 0.0404$). However, Cohen’s $d$ of $- 1.9775$ indicates a very large effect size, suggesting that vein tortuosity is significantly greater than artery tortuosity.

In contrast, the LES-AV glaucoma data showed lower mean tortuosity: $0.9791$ for arteries and $0.9936$ for veins. The distribution of arterial tortuosity deviated significantly from normality (Shapiro-Wilk $p = 0.0157$), leading to a non-parametric analysis. A moderate negative Spearman correlation ( $r = - 0.6402,\; p = 0.0338$) was observed, suggesting an inverse relationship between arterial and venous tortuosity. However, the Mann-Whitney U test ( $p = 0.642$) and Wilcoxon signed-rank test ( $p = 0.5488$) revealed no statistically significant difference between artery and vein tortuosity.

**Figure 13:**
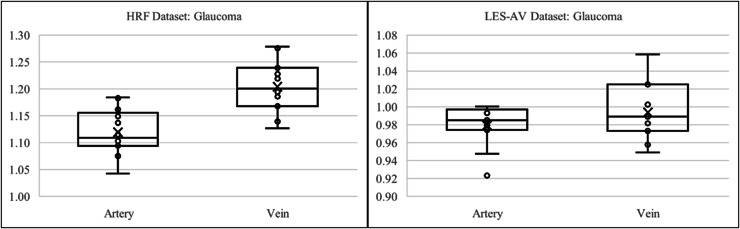
Boxplot comparison of artery and vein tortuosity in glaucomatous eyes from HRF and LES-AV datasets. HRF images (FOV: 45°) show a distinct difference between artery and vein tortuosity, while LES-AV images (FOV: 30°) show more overlapping distributions. This supports the statistical outcomes in Table 6 and highlights the impact of imaging characteristics on tortuosity analysis.

Figure 13 illustrates the boxplot comparison of artery and vein tortuosity in glaucomatous eyes across two datasets with different imaging characteristics. In the HRF dataset, the tortuosity of veins is markedly higher than that of arteries, consistent with the large effect size indicated in Table 6. This difference is visually evident in the wider interquartile range and elevated median of the vein group. In contrast, the LES-AV dataset shows a narrower range and overlapping distributions for arteries and veins, reflecting the absence of statistically significant differences. These visual patterns reinforce the findings in Table 6 and emphasize the influence of field of view and dataset characteristics on tortuosity measurements in diseased eyes.

**Figure 14:**
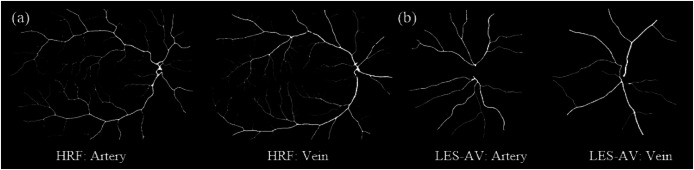
Samples of segmented artery and vein tortuosity of glaucomatous eyes in the HRF and LES-AV datasets. In the HRF dataset, the tortuosity of veins is markedly higher than that of arteries. Meanwhile in the LES-AV dataset shows a narrower range and overlapping distributions, reflecting the absence of statistically significant differences. (A) HRF sample for artery and vein (FOV: 45°). (B) LES-AV sample for artery and vein (FOV: 30°).

Figure 14 shows samples of segmented artery and vein of glaucomatous eyes in the HRF and LES-AV datasets.

## Discussion

This section interprets the findings on retinal vessel tortuosity obtained across multiple datasets, vessel types, and disease conditions. The discussion aims to contextualize the observed variations in tortuosity between arteries and veins, between healthy and diseased eyes, and across datasets with different imaging characteristics.

### Artery-vein tortuosity patterns in healthy eyes under varying field of view (FOV) settings

The first analysis explores tortuosity variation between arteries and veins, in healthy eyes, and the impact of imaging datasets on tortuosity measurements.

Across all three datasets, veins consistently exhibit higher tortuosity values and greater variability than arteries which is supported by Giesser et al. (2024). While DRIVE dataset suggests moderate correlation between artery and vein tortuosity, HRF and LES-AV reveal little to no relationship. This indicates that artery and vein tortuosity are largely independent features, influenced by different physiological or structural factors. The consistently large effect sizes (Cohen’s $d > 1$) confirm that artery-vein tortuosity differences are not only statistically significant but also practically meaningful. These findings support the handling of artery and vein tortuosity as separate variables. In summary, this analysis demonstrates that:

- Veins are significantly more tortuous than arteries in normal retinal images.
- The correlation between artery and vein tortuosity is dataset-dependent and generally weak.
- Despite variation in correlation, the magnitude of difference between vessel types remains robust.

This highlights the need for artery-vein differentiation in tortuosity-based diagnostic approaches.

The second analysis investigates the impact of dataset imaging characteristics such as field of view (FOV) on tortuosity measurements in healthy eyes. Effect sizes calculated using Cohen’s d show substantial differences between arteries and veins across all datasets: –2.4707 (HRF), –1.5859 (DRIVE), and –1.0112 (LES-AV). Again, these results confirm that veins are consistently more tortuous than arteries, regardless of dataset.

Overall, this analysis highlights that dataset characteristics especially FOV, significantly influence tortuosity measurements. While the trend of higher vein tortuosity is consistent, the absolute values differ depending on the dataset, indicating the need for FOV standardization in comparative retinal studies. While the comparison across datasets primarily highlights differences in FOV (with DRIVE and HRF employing a 45° FOV and LES-AV using a narrower 30° FOV) it is also important to consider the impact of image orientation on tortuosity measurement. The DRIVE and HRF datasets typically feature images with left or right eye orientations, where the optic disc is positioned nasally. In contrast, the LES-AV dataset presents centered images with the optic disc located closer to the center of the frame. These orientation and positioning differences, when combined with FOV variations, can significantly influence the visibility and traceability of peripheral vessels. This is particularly relevant in narrower FOV images, where peripheral structures may be underrepresented. As such, variability in tortuosity measurements may not be attributed to FOV alone, but also to image acquisition geometry and field positioning. This observation aligns with previous findings that wider FOVs facilitate the capture of peripheral retinal features, which are essential for comprehensive and accurate tortuosity assessment (Ramos et al., 2020).

### Artery-vein tortuosity patterns in healthy and diseased eyes (HRF dataset)

The second analysis examines whether pathological conditions specifically glaucoma and diabetic retinopathy (DR) affect vessel tortuosity, and whether such changes are consistently observed in both arteries and veins. The analysis is based on the HRF dataset.

Overall, in both glaucoma and DR cases, veins consistently show higher tortuosity than arteries, which is the same pattern found in healthy eyes. However, the degree of difference is more pronounced in DR, indicated by a higher Cohen’s $d$ and mean separation. Correlation between artery and vein tortuosity remains weak across all conditions. These observations suggest that while tortuosity alone may not reflect direct vessel-to-vessel relationships, it does highlight disease-specific vascular alterations, especially in DR as supported by Zhang & Deng (2024). Therefore, tortuosity analysis remains a valuable non-invasive biomarker for distinguishing between normal and pathological retinal states.

### Artery-vein tortuosity patterns in healthy and glaucoma eyes (LES-AV dataset)

This analysis extends the evaluation of disease-related tortuosity changes by using the LES-AV dataset to compare healthy and glaucomatous eyes. It examines whether glaucoma leads to significant alterations in vessel tortuosity and whether those changes manifest differently in arteries *vs*. veins.

It was found that the glaucoma eyes in the LES-AV dataset exhibit reduced tortuosity in both arteries and veins compared to healthy eyes. The results showed that the typical distinction between artery and vein tortuosity observed in healthy cases diminishes in glaucomatous eyes, suggesting that disease-induced vascular remodeling may reduce the difference. The presence of a significant inverse correlation in glaucoma further supports altered vessel dynamics in disease states. Similar results was also obtained by Badawi et al. (2023) when their study showed that tortuosity in artery and vein from DRIVE, STARE (FOV: 35°) and CHASEDB (FOV: 30°) datasets has no significant difference.

Since the LES-AV dataset has a narrower field of view (30°) and a centered optic disc orientation, in contrast to datasets like HRF where the optic disc typically appears in a nasal position (left or right side), these imaging characteristics may limit the visibility of peripheral vessels and influence tortuosity quantification, particularly in disease cases where vessel branching and curvature are less obvious. Therefore, caution must be taken when interpreting the reduced artery-vein tortuosity differences in glaucomatous eyes, as they may partially result from dataset acquisition properties rather than true physiological changes alone.

### Artery-vein tortuosity patterns in diseased eyes under varying field of view (FOV) settings (HRF and LES-AV)

In the final analysis, we assess how dataset characteristics influence tortuosity measurements in glaucomatous eyes. Comparing results from HRF and LES-AV datasets, the goal is to determine whether the FOV affects the magnitude or pattern of tortuosity variation in disease conditions.

It can be concluded that while the HRF dataset shows a clear and significant difference between arterial and venous tortuosity in glaucomatous eyes, the LES-AV dataset does not. These differences may stem from imaging protocols *i.e*., the FOV. The findings emphasize the impact of dataset characteristics on retinal tortuosity analysis, particularly in disease studies. Thus, researchers should consider for dataset-specific biases when designing or comparing automated diagnostic models based on tortuosity features.

Additionally, the orientation of the optic disc within the images may influence tortuosity measurements. The HRF images typically present the optic disc in a nasal (left or right) orientation, and LES-AV images are centered around the optic disc. This orientation difference affects the extent of visible peripheral vessels, particularly those with higher curvature which can in turn influence the calculated tortuosity values. In LES-AV images, this central view may limit peripheral vessel visibility, potentially explaining the reduced artery-vein tortuosity difference observed in glaucomatous eyes from that dataset.

Our artery–vein separation currently depends on RGB ground-truth labels in which the datasets without these annotations cannot be processed for vessel-specific tortuosity, limiting the applicability and cross-dataset generalizability. As future work, we aim to replace this dependency with an automated artery–vein classifier or semi-automatic labeling to enable use on unlabeled clinical images.

## Conclusion

This study investigated the behavior of retinal vascular tortuosity through automated analysis using three publicly available fundus image datasets: DRIVE, HRF, and LES-AV. Each dataset is with distinct imaging parameters and anatomical orientations. By analyzing arteries and veins across healthy, glaucomatous, and diabetic retinopathy (DR) eyes, the research offers a comprehensive assessment of tortuosity as a potential biomarker of retinal vascular health.

Across all datasets, veins consistently exhibited higher tortuosity than arteries in healthy eyes, a finding supported by large effect sizes (Cohen’s $d$), despite weak and inconsistent correlation between the two vessel types. These results suggest that artery and vein tortuosity are largely independent structural features, potentially governed by different physiological factors. DR cases showed even greater tortuosity separation between arteries and veins, indicating disease-induced vascular remodeling. In contrast, glaucomatous eyes presented more complex patterns: although the HRF dataset maintained a clear artery-vein distinction, the LES-AV dataset showed weaker separation, likely due to both non-normal data distributions and imaging constraints.

A critical insight from this study is the influence of dataset-specific imaging characteristics, particularly FOV and optic disc orientation, on tortuosity measurements. DRIVE and HRF datasets, with a wider FOV (45°) and a nasal optic disc placement (OD on the left or right), captured more peripheral vessels, which are typically more tortuous. This contributed to higher overall tortuosity values. In contrast, the LES-AV dataset, with a narrower FOV (30°) and a centered optic disc, offered limited visibility of peripheral vessels, leading to lower tortuosity values and reduced artery-vein distinction. These anatomical and technical factors can significantly affect tortuosity outcomes and must be carefully considered when comparing or generalizing results across datasets.

In summary, this study demonstrates that:

- Tortuosity differs significantly between arteries and veins, even under normal conditions.
- Disease states, such as glaucoma and diabetic retinopathy, amplify or alter tortuosity behavior in distinct ways.
- Imaging parameters especially FOV and optic disc orientation, strongly influence tortuosity measurements and should be standardized or adjusted for in comparative retinal studies.

Overall, these findings validate retinal tortuosity as a robust, vessel-specific indicator of vascular morphology with diagnostic potential. This also indicate the need for anatomically aware image analysis approaches to ensure accurate and standardized assessments in future clinical and AI-assisted diagnostic models.

## Supplemental Information

10.7717/peerj.20561/supp-1 Supplemental Information 1 MATLAB Code for Retinal Vessel Tortuosity Measurement.

## Additional Information and Declarations

### Data Availability

The following information was supplied regarding data availability:

The tortuosity code is available in the Supplemental File.
